# ASSESSMENT OF EARLY INDICATORS FOR SEPSIS DEVELOPMENT IN MULTIPLE TRAUMA PATIENTS—THE SEPSIS AS TRAUMA OUTCOME PREDICTION (STOP) SCORE

**DOI:** 10.1097/SHK.0000000000002626

**Published:** 2025-05-14

**Authors:** Nils Becker, Jasmin Maria Bülow, Niklas Franz, Ingo Marzi, Florian Gebhard, Akiko Eguchi, Helen Rinderknecht, Borna Relja

**Affiliations:** 1 Department of Trauma, Hand, Plastic and Reconstructive Surgery, Translational and Experimental Trauma Research, Ulm University Medical Center, Ulm, Germany; 2 Department of Trauma, Hand and Reconstructive Surgery, Goethe University Frankfurt, Frankfurt, Germany; 3 Department of Gastroenterology and Hepatology, Mie University Graduate School of Medicine, Tsu, Japan

**Keywords:** Extracellular particles, vesicles, scoring, inflammation, prediction, polytrauma, risk assessment, AIS—Abbreviated Injury Scale, APACHE—Acute Physiology and Chronic Health Evaluation, ATLS—advanced trauma life support, AUC—area under the ROC curve, CI—confidence intervals, CRP—c-reactive protein, CT—computed tomography, DAMP—damage-associated molecular patterns, ED—emergency department, EP—extracellular particle, EV—extracellular vesicles, FFP—fresh frozen plasma, Fig—figure, ICU—intensive care unit, IL—interleukin, IQR—interquartile range, INR—international normalized ratio, ISS—Injury Severity Score, MISEV—minimal information for the study of extracellular vesicles, MODS—multiorgan dysfunction syndrome, PCT—procalcitonin, PPV—positive predictive value, PRBC—packed-red blood cell, SBP—systolic blood pressure, SEM—standard error of the mean, SIRS—systemic inflammatory response syndrome, SOFA—sequential organ failure assessment, STOP—sepsis as trauma outcome prediction, STROBE—Strengthening the Reporting of Observational Studies in Epidemiology, ROC—receiver operating characteristic, Tab—table, TPT—thromboplastin time, TSS—traumatic sepsis score, U—units

## Abstract

**Background:** Infections are common complications in critical care, particularly in patients with severe multiple trauma, who are at elevated risk due to trauma-induced immunological changes. The heterogeneity of trauma patients complicates their initial assessment, yet timely recognition of patients at risk is crucial for guiding therapy and preventive measures. This study evaluated risk factors for sepsis and pneumonia in multiple trauma patients, incorporating a novel parameter: cell-derived extracellular particles (EPs) in plasma. **Methods:** Severely injured multiple trauma patients aged 18–80 years with an Injury Severity Score (ISS) ≥16 were included. Patient- and injury-related parameters were assessed at the injury site, admission and during clinical course. EP counts in plasma were measured at admission using intravesicular staining. Key variables from the first 24 h were analyzed to develop an early risk assessment score. **Results:** Among 124 patients, 16 developed pneumonia, and 29 developed sepsis. Pneumonia was associated with significantly lower Glasgow Coma Scale scores, higher intubation rates at the injury site and elevated Sequential Organ Failure Assessment scores at admission. Sepsis correlated with higher ISS, increased 24-h transfusion rates, lower leukocyte counts on day 1, and decreased levels of small EPs in plasma at admission. These variables formed the weighted Sepsis as Trauma Outcome Prediction (STOP) score. A STOP score >3 had a positive predictive value of 59.4% within 24 h upon admission to the emergency department for subsequent sepsis development. **Conclusion:** The risk of pneumonia in severely injured trauma patients was linked to impaired consciousness and preexisting organ-dysfunctions at admission. High-risk sepsis patients could be identified on day 1 following trauma using the STOP score, which incorporates ISS, 24-h transfusion rates, leukocyte counts at day 1, and small EP rates at admission. This novel scoring system could facilitate targeted therapeutic and preventive strategies for distinguishing high-risk populations.

## INTRODUCTION

The prevention, early recognition, and treatment of infections in critically ill patients remain challenging and will potentially become even more difficult, given the increasing multimorbidity of patients and multidrug resistance of pathogens (1). Severely injured trauma patients are especially vulnerable to infections, due to their specific immunologic responses triggered by trauma-induced tissue damage (2,3). For instance, severe blunt trauma is associated with genomic changes in leukocyte fractions (4), creating an immune environment conducive to complications caused by invading pathogens and, the development of sepsis.

Sepsis, defined as infection-triggered, rapidly progressing organ dysfunction (5), is associated with high rates of mortality, while sepsis survivors often endure severe long-lasting complications that significantly impair their quality of life (6,7). Although advancements in critical care have reduced mortality rates associated with sepsis and multiorgan dysfunction syndrome (MODS) in trauma patients over the last decades (8), the incidence of sepsis among multiple-trauma patients has remained consistent, affecting 10%–30% of severely injured patients (9–11). The persistent prevalence of sepsis, the increasing complexity of pathogens, the substantial impact on patient outcomes, and patient’s quality of life with tremendous socioeconomic impact (12), highlight the urgent need for improved methods to identify and manage the high-risk population of critically ill trauma patients who might develop sepsis.

Therefore, early recognition of multiple trauma patients at risk of infections is critical for the accurate timing of interventions and providing individualized, risk-adapted therapies. Identifying at-risk patients could also enable the implementation of experimental prophylactic approaches and lead to more homogenous study populations for future research.

However, distinguishing between trauma-associated and infection-associated inflammation remains particularly demanding, as both can result in organ dysfunction (13,14).

Given the tremendous trauma-induced cellular genomic alterations, in this study, we aimed to enhance the early risk assessment by incorporating cell-derived factors into the initial evaluation of severely injured patients. Therefore, we focused on the extracellular particle (EP) rate in patient’s plasma at admission in combination with established clinical markers, to evaluate their prognostic value for pneumonia and sepsis development. Previous research demonstrated a significant correlation between elevated rates of size-defined, cell-derived EPs in multiple trauma patients and in-hospital mortality (15). Building on this promising data, we hypothesized that EP rates could serve as a promising marker for predicting pneumonia and sepsis in multiple trauma patients.

By investigating these early biomarkers, this study aims to contribute to more effective risk stratification and targeted interventions for critically ill trauma patients.

## METHODS

### Ethics and patient enrollment

This study was performed with the approval of the local ethics committee and in accordance with the STROBE guidelines (16) and the Declaration of Helsinki at the University Hospital of the University Ulm and at the Goethe University Hospital Frankfurt (number of the ethical approval: 312/10). Informed consent was obtained from all included patients or their nominated legally authorized representative.

All patients were admitted to the level one trauma center. Inclusion criteria were an acute traumatic injury affecting more than one body region with an Injury Severity Score (ISS) ≥16 and age between 18 and 80 years. Patients with preexisting anticoagulative or immunosuppressive medication, immunologic disorder, concomitant acute myocardial infarction, thromboembolic events, or burns and patients with immediate death after admission were excluded. All patients were treated according to the latest advanced trauma life support protocol and in accordance with the German national guidelines for the treatment of severely injured patients. If indicated, patients received a whole body computed tomography (CT) scan. The final radiological report provided by the radiologist as part of the initial trauma assessment team, allowed rapid calculation of the ISSs within the first 24 h after admission as described below. All injuries were assessed by using the Abbreviated Injury Scale (AIS) (17) in the according body region. A severe injury in a body region was defined by an AIS of ≥3. The ISS was calculated out of the single AIS scores as described before (18). The Sequential Organ Failure Assessment (SOFA) score (5) and the Acute Physiology and Chronic Health Evaluation (APACHE) II score (19) at admission were calculated as described using the physiological and laboratory variables recorded during the emergency treatment. The rate of operative emergency procedures was assessed retrospectively, including invasive treatments as emergency laparotomy, craniotomy, (external) fracture fixation, or emergency spinal decompression and stabilization. Minimal-invasive procedures as superficial wound closure, intracranial pressure probe implantation, or chest tube insertion were not included.

### Endpoint definition

For the statistical analysis in-hospital mortality and the development of pneumonia and sepsis were assessed, as defined by the treating physician during the clinical course. All clinical data and routine laboratory parameters were assessed by using the medical record during the hospital stay and the discharge letter. The diagnose of pneumonia was defined by clinical, radiologic, and bacteriologic findings including new pulmonary infiltrates on chest X-ray as well as one of the following criteria: positive blood culture, bronchial alveolar lavage, and/or sputum culture (20). The initial assessment of sepsis in this study adhered to the 2005 criteria established by the International Sepsis Forum, which defined sepsis based on the fulfillment of the systemic inflammatory response syndrome (SIRS) criteria in conjunction with a confirmed infection (20). A retrospective reclassification of sepsis was conducted using the Sepsis-3 criteria (5). Of note, patient records that provided a single daily recording of the worst SOFA score over a 10-day period following hospital admission were applied. The classification under the Sepsis-3 criteria was based exclusively on changes in SOFA score of ≥2 points within a 24-h timeframe. All patients categorized as septic according to old definition fulfilled the Sepsis-3 criteria. Patients that developed a pneumonia with sepsis were only included in the sepsis cohort.

### Blood sampling

Pre-chilled Ethylenediaminetetraacetic acid sample tubes (BD vacutainer, Becton Dickinson Diagnostics, Aalst, Belgium) were used for blood sampling directly upon admission in the emergency department. The samples were immediately stored on ice and subsequently centrifuged at 2000 x g for 15 min at 4°C. The obtained plasma was stored at −80°C until the EP analysis and cytokine measurements. Despite the prospective inclusion of patients, the samples and clinical data were analyzed retrospectively.

### Extracellular particle staining

The EP rate was assessed as described before (15). Briefly, the stored plasma was thawed and immediately stained using 4 μg/mL Calcein-AM staining (Invitrogen, San Diego, CA) for 30 min at room temperature in the dark. Stained particles were analyzed by flow cytometry (BD Canto II; BD Biosciences, San Jose, CA). UV-conjugated alignment beads sizing 2.5 μm (Life Technologies) were used as size standard. The size of the particles was assessed using standardized size-differentiation beads (Spherotech nano fluorescent particle size standard kit (Spherotech, Lake Forest, IL) and FluoSpheres biotin-labeled 0.04 μm yellow-green (Life Technologies). Validation of the intravesicular staining by this method has been shown before (15,21). In accordance with the recent minimal information for the study of extracellular vesicles (MISEV) 2023 (22), discriminating small and large extracellular vesicles, the analysis cutoff of 200 nm was applied.

### Cytokine-measurement of the plasma samples

The cytokines levels of interleukin (IL)-6 and IL-10 were measured in the plasma using IL-6 and IL-10 Eli-pair ELISA-Assay (Diaclone, Hoelzel Diagnostica, Cologne, Germany) according to the manufacturer’s recommendations.

### Development of a clinically implementable risk-scoring

To identify patients at risk for sepsis or pneumonia, we clustered early assessable variables in four different categories. The categories were included in a risk scoring, if one variable displayed a significant correlation with sepsis or pneumonia development. If more than one marker in each category correlated significantly, only one marker out of each category was chosen based on clinical accessibility and the prognostic value. Cut-Offs for significant parameters were chosen based on a high sensitivity with an adequate specificity, as the focus of the score lies in an early screening of potential patients. We defined patient- and injury characteristics (age, sex, ISS, AIS, GCS [Glasgow Coma Scale], SOFA, APACHE-II, intubation rate, emergency operation rate), hemorrhage and coagulation characteristics (systolic blood pressure, shock index, heart rate, mean arterial blood pressure, heart rate, packed-red blood cell [PRBC] transfusion, fresh frozen plasma [FFP], volume transfusion, hemoglobin level, thromboplastin time [TPT], partial thromboplastin time, international normalized ratio [INR], fibrinogen, platelet count, pH, lactate), inflammatory markers (leukocyte count, c-reactive protein [CRP], IL-6, IL-10 procalcitonin [PCT]), and direct cell-derived parameters (EP count) as categories.

### Statistical analysis

The dataset was evaluated for normality using the Kolmogorov-Smirnov test with the Dallal-Wilkinson-Lilliefor correction. Group differences were assessed using the unpaired non-parametric Mann-Whitney *U* test. Time-course analyses of daily values, compared to baseline levels measured in the emergency department (ED) within the same groups, were evaluated using the non-parametric Wilcoxon matched-pairs signed rank test. Proportional analyses were conducted with the chi-square test. Receiver operating characteristic (ROC) curves were generated to determine optimal cutoff values, with 95% confidence intervals (CIs) calculated using the Clopper-Pearson method. Non-parametric two-tailed Spearman correlation was used to compute the Spearman correlation coefficient (r) along with 95% CI and *P* values. The area under the ROC curve (AUC), as well as sensitivity and specificity, were assessed. Data are reported as mean ± standard error of the mean (SEM) unless specified otherwise. Furthermore, a logistic regression analysis was performed to evaluate the association between clinical and laboratory parameters and the likelihood of sepsis. The binary outcome variable was group by status (0 = no sepsis, 1 = sepsis). Predictor variables included: small EPs, ISS, PRBC, and leukocyte count. Prior to modeling, the dataset was screened for missing values, and rows with incomplete data for any of the predictor variables were excluded. A multivariable logistic regression model was then fitted using the maximum likelihood estimation method. Wald tests were used to assess the statistical significance of individual predictors in the model. Coefficients were exponentiated to derive odds ratios (ORs) along with their 95% confidence intervals (CIs), providing a measure of effect size. The analyses were conducted using Python (version 3.10) with the statsmodels library (version 0.14.0). A *P* value of <0.05 was considered statistically significant. Statistical analyses were conducted using GraphPad Prism 10.0 software (GraphPad Software Inc., San Diego, CA).

## RESULTS

### General patient and injury characteristics

In total, 1,556 patients were screened. Of these, 318 patients initially met the inclusion criteria. Among them, 53 patients had their ISS corrected within 24 h and were subsequently excluded, along with the removal of their blood samples. This left 265 patients meeting the inclusion criteria. Thirteen patients who died immediately were also excluded, resulting in 252 remaining patients. Blood sampling was missed in 33 of these cases, and for additional 95 patients, informed consent for study participation could not be obtained, resulting in a total study population of 124 patients.

Of the included 124 patients, 79 were analyzed in the no complication cohort, 16 in the pneumonia cohort and 29 in the sepsis cohort. No significant difference of age and sex between the groups was observed.

The median ISS was significantly higher in the sepsis cohort compared to the no complication cohort (34 [interquartile range {IQR} 23.5–38.5] vs. 24 [IQR 18–29], *P* < 0.05, Suppl. Table 1, http://links.lww.com/SHK/C448). In addition, patients who developed sepsis had a higher rate of severe chest injures compared to the no complication and pneumonia cohort (68.97% vs. 46.84% vs. 37.50%, *P* < 0.05, Suppl. Table 1, http://links.lww.com/SHK/C448) and higher rates of severe abdominal injuries compared to the no complication group (31.03% vs. 10.13%, *P* < 0.05, Suppl. Table 1, http://links.lww.com/SHK/C448).

Patients who developed pneumonia or sepsis stayed longer on the intensive care unit (ICU) compared to patients in the no complication cohort (12.60 ± 1.98 days and 19.03 ± 3.47 days vs. 7.34 ± 0.97 days, *P* < 0.05, Suppl. Table 1, http://links.lww.com/SHK/C448). The length of the hospital stay was significantly longer in the sepsis cohort compared to no complication and the pneumonia cohort (36.72 ± 4.89 days vs. 18.08 ± 1.87 days and 18.56 ± 2.95 days, *P* < 0.05, Suppl. Table 1, http://links.lww.com/SHK/C448). In total, 16 patients died with no significant differences between the groups. The rate of early invasive operative treatments trended to be higher in the sepsis cohort (22/29 patients; 75.9%, Suppl. Table 1, http://links.lww.com/SHK/C448), compared to the no complication (48/79 patients; 60.8%, Suppl. Table 1, http://links.lww.com/SHK/C448, *P* = 0.1452) and the pneumonia cohort (10/16 patients; 62.5%, Suppl. Table 1, http://links.lww.com/SHK/C448, *P* = 0.3438) with no significant differences. A full overview of the assessed patient and injury characteristics is provided in the supplementary Table 1.

### Parameters assessed in the field and upon admission

Upon admission to the emergency department, patients who developed sepsis had a significantly higher shock index compared to those in the no complication cohort (0.85 ± 0.07 vs. 0.71 ± 0.04, *P* < 0.05, Suppl. Table 2, http://links.lww.com/SHK/C449). The pneumonia cohort displayed a significantly lower GCS both in the field and at emergency department admission compared to the sepsis and no complication cohorts (field: 3 [3–12.25] vs. 14 [3.5–15] vs. 13 [6–15], *P* < 0.05; admission: 3 [3] vs. 3 [3–15] vs. 6 [3–14.75], *P* < 0.05, Suppl. Table 2, http://links.lww.com/SHK/C449).

FFP transfusion rates within the first 24 h and cumulatively in total were significantly elevated in the sepsis cohort compared to the no complication and pneumonia groups (24 h: 4.64 ± 1.67 units vs. 1.77 ± 1.05 units vs. 0.38 ± 0.38 units, *P* < 0.05; total: 5.29 ± 1.78 units vs. 1.74 ± 1.04 units vs. 0.38 ± 0.38 units, *P* < 0.05, Suppl. Table 2, http://links.lww.com/SHK/C449). PRBC transfusion units in the first 24 h were significantly higher in the sepsis cohort than in the no complication and pneumonia cohorts (5.36 ± 1.70 units vs. 2.48 ± 1.14 units vs. 1.50 ± 0.80 units, *P* < 0.05, Suppl. Table 2, http://links.lww.com/SHK/C449). Total PRBC transfusion units were also significantly higher in sepsis patients compared to the no complication cohort (8.75 ± 2.29 units vs. 3.55 ± 1.14 units, *P* < 0.05, Suppl. Table 2, http://links.lww.com/SHK/C449).

Patients in the sepsis group received a significantly larger volume of fluid transfusion than those to the no complication cohort (1102.00 ± 100.10 mL vs. 900.60 ± 88.45 mL, *P* < 0.05, Suppl. Table 2, http://links.lww.com/SHK/C449). The INR, reflecting coagulation status, was significantly elevated in the sepsis cohort compared to the no complication group (1.188 ± 0.037 vs. 1.144 ± 0.035, *P* < 0.05, Suppl. Table 2, http://links.lww.com/SHK/C449). Pneumonia cohort exhibited significantly higher SOFA score at admission compared to the no complication cohort (8 [5.5–9.0] vs. 2 [1.0–7.0], *P* < 0.05, Suppl. Table 2, http://links.lww.com/SHK/C449), along with significantly elevated APACHE-II Score (22 [16.00–23.00] vs. 11 [5.00–18.75], *P* < 0.05, Suppl. Table 2, http://links.lww.com/SHK/C449). The intubation rate on field was significantly increased in the pneumonia cohort compared to the no complication cohort (75% vs. 40.5%, *P* < 0.05, Suppl. Table 2, http://links.lww.com/SHK/C449), and in the emergency department increased in the pneumonia cohort compared to the no complication and the sepsis cohort (100% vs. 54.4% vs. 72.4%, *P* < 0.05, Suppl. Table 2, http://links.lww.com/SHK/C449). No significant difference was observed between the sepsis and no complication cohort. A full overview of the assessed physiological and laboratory characteristics is provided in supplementary Table 2.

### Decreased rate of circulating EPs in sepsis patients at admission

**Fig. 1:**
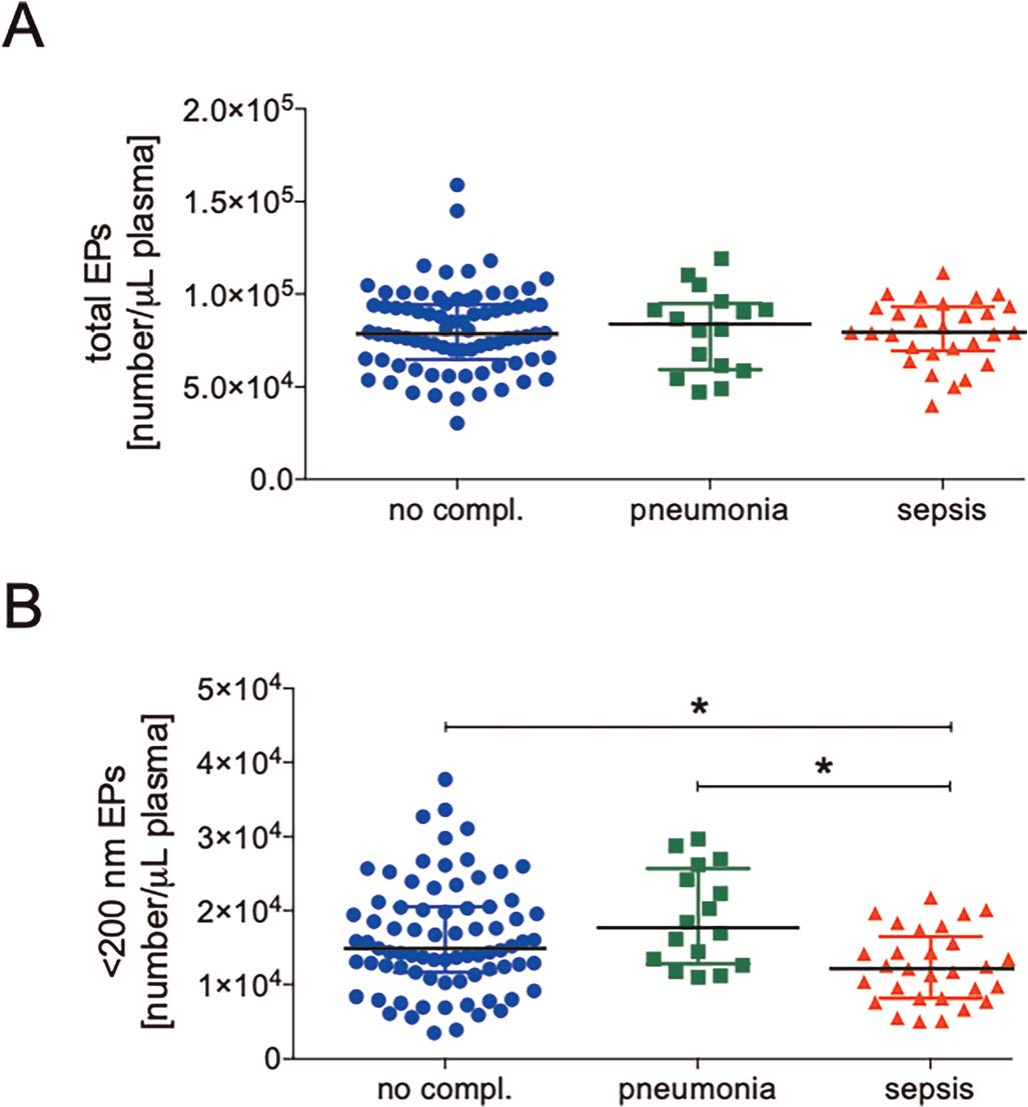
**Rates of total circulating EPs (A) and EPs under 200 nm (B) upon admission in three study cohorts: no complications (no compl.), pneumonia and sepsis**. Data is presented as individual rates of each patient according to their study cohort and the group median with interquartile range. Significant differences with a *P* < 0.05 are indicated with *. EPs, extracellular particles.

The total EP rate did not differ significantly between the groups (Fig. 1A). However, patients who developed sepsis had a significantly lower rate of EPs < 200 nm in plasma at admission compared to the pneumonia and no complication cohorts (*P* < 0.05, Fig. 1B).

To further investigate the prognostic potential of the EP rate across patient cohorts, additional analyses were performed (Suppl. Fig. 1, http://links.lww.com/SHK/C456 and Table 1). The diagnostic performance of EPs < 200 nm was evaluated for different patient cohorts using ROC curves. For the no complications cohort, the AUC was 0.5660 (95% CI: 0.4621–0.6698, Suppl. Fig. 1A, http://links.lww.com/SHK/C456), with a *P* value of 0.2230, indicating no statistically significant diagnostic utility. The optimal cutoff value was >17.380 EPs/μL, with a sensitivity of 40.51% (95% CI: 29.60–52.15), specificity of 68.89% (95% CI: 53.35–81.83), and a likelihood ratio of 1.302 (Table 1).

**Table 1:** Cutoff and prognostic values for the rates of circulating EPs upon admission to distinguish patients who developed no complications, pneumonia, or sepsis after trauma

Parameters	Cutoff value	Sensitivity % (95% CI)	Specificity % (95% CI)	AUC (95% CI)	Likelihood ratio	*P* < 0.05
**<200 nm EPs (number/μL) no complications**	>17,380	40.51 (29.60–52.15)	68.89 (53.35–81.83)	0.5660 (0.4621–0.6698)	1.302	0.2230
**<200 nm EPs (number/μL) pneumonia**	<16,080	56.96 (45.33–68.06)	62.50 (35.43–84.80)	0.6044 (0.4609–0.7479)	1.519	0.1893
**<200 nm EPs (number/μL) sepsis**	>12,639	69.62 (58.25–79.47)	58.62 (38.94–76.48)	0.6600 (0.5516–0.7683)	1.682	0.0111

AUC, area under the receiver operating characteristic curve; CI, confidence interval; EPs, extracellular particles.

In the pneumonia cohort, the AUC was 0.6044 (95% CI: 0.4609–0.7479, Suppl. Fig. 1B, http://links.lww.com/SHK/C456), with a *P* value of 0.1893, reflecting modest discriminatory capacity. The optimal cutoff value was <16.080 EPs/μL, yielding a sensitivity of 56.96% (95% CI: 45.33–68.06), specificity of 62.50% (95% CI: 35.43–84.80), and a likelihood ratio of 1.519 (Table 1).

For the sepsis cohort, the AUC was 0.6600 (95% CI: 0.5516–0.7683, Suppl. Fig. 1C, http://links.lww.com/SHK/C456), with a significant *P* value of 0.0111. The optimal cutoff value was >12.639 EPs/μL, with a sensitivity of 69.62% (95% CI: 58.25–79.47), specificity of 58.62% (95% CI: 38.94–76.48), and a likelihood ratio of 1.682 (Table 1). A full overview of different cutoffs for sepsis is available in supplementary Table 3A (Suppl. Table 3A, http://links.lww.com/SHK/C450). These findings indicate that EPs < 200 nm has potential utility as an early diagnostic marker for sepsis but limited diagnostic value for the no-complications and pneumonia cohorts.

### Course of inflammatory and clinical markers

**Fig. 2:**
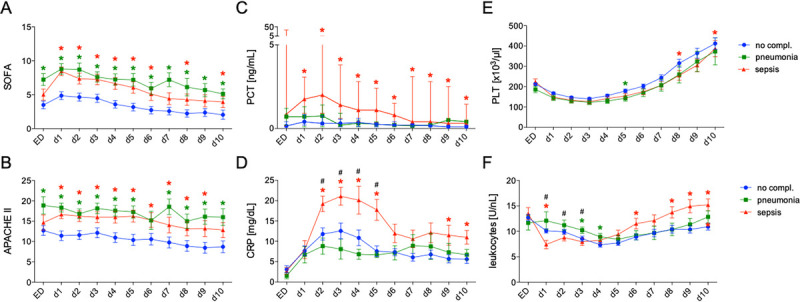
**Changes in clinical and laboratory parameters upon admission to the ED over 10 days daily (d) in patients without complications (no compl., blue), with pneumonia (green), and with sepsis (red)**. A, SOFA scores, APACHE-II scores (B), PCT (C) levels (median and interquartile range), CRP levels (D), PLTs (E), and leukocyte counts (F) are shown. Significant differences with a *P* value <0.05 are given as following: * denotes differences between sepsis or pneumonia cohort *versus* no complication cohort; # indicates differences between sepsis *versus* pneumonia cohort. APACHE-II, Acute Physiology and Chronic Health Evaluation II; CRP, C-reactive protein; ED, emergency department; PCT, procalcitonin; PLT, platelet ; SOFA, Sequential Organ Failure Assessment;

Assessment of the SOFA score over the first days revealed a significantly higher admission score in the pneumonia cohort compared to the sepsis and no complication cohorts. The SOFA score remained consistently significantly elevated in the pneumonia group compared to the no complication group across all time points. In the sepsis cohort, a rapid increase in the SOFA score was observed after the first day, reaching levels similar to the pneumonia cohort. Scores in all groups subsequently decreased continuously, although the sepsis and pneumonia cohorts maintained significantly elevated scores compared to the no complication cohort from days 1 to 6 posttrauma (Fig. 2A).

A similar pattern was observed for the APACHE-II score (Fig. 2B). PCT levels were comparable across groups at admission, but significantly increased only in the sepsis cohort from day 1 posttrauma compared to the no complication group, remaining significantly elevated throughout the observational period (Fig. 2C). PCT peaked on day 2 posttrauma, followed by a continuous decrease. Comparable trends were observed for CRP levels, which showed a significant increase only in the sepsis cohort on day 2, compared to the pneumonia and no complication cohorts (Fig. 2D). CRP levels peaked on day 3, with a rapid decline between days 5 and 6. While the pneumonia and no complication groups also demonstrated lower CRP levels, the pneumonia group exhibited a trend toward a second peak at day 7 posttrauma, without significant differences from the other cohorts.

Total platelet counts showed similar trends across all groups, showing an initial and continuous decrease up to day 3, followed by a steady increase. The no complication cohort displayed a more pronounced rise, with significantly higher platelet counts compared to the pneumonia cohort on day 5 and the sepsis cohort on days 8 and 10 (Fig. 2E). Total leukocyte counts in the sepsis cohort showed a significant decrease on day 1 posttrauma compared to the no complication and pneumonia cohorts. From day 1 onward, leukocyte counts in the sepsis cohort increased continuously, remaining significantly increased compared to the no complication group on day 6 and 8–10. A milder initial decrease in total leukocyte counts was also observed in the no complication and pneumonia cohorts, followed by a trend toward increases on days 4 and 5 (Fig. 2F).

### Assessment of the diagnostic value of significant markers for the development of a risk scoring

Only limited parameters showed significant differences between the pneumonia cohort and the other cohorts. These parameters were not accessible for a valid risk scoring with useful power.

For sepsis development, the prognostic value of easy assessable parameters analyzed within the first 24 h after admission, with significant differences between the sepsis and no complication cohorts, was evaluated. The included parameters were the ISS, PRBC transfusion rate within the first 24 h, the <200 nm EP rate at admission, and the leukocyte count on day 1 after admission (Table 2). ISS has been chosen, as it is an assessable value, containing the main information about the overall injury severity, including all AIS regions. Despite its known limitations, such as interobserver variance, correlations between sepsis development and ISS have been described before (23). In addition, in our study, the ISS correlated with the rate of emergency surgeries (Spearman r = 0.1872 (95% CI: 0.0045–0.3579), *P* = 0.0389), why we did not additionally include the emergency operation rate. PRBC transfusion rate has been associated with sepsis and complication development before (23,24), therefore and because of the easily assessable values in the clinical setting PRBC transfusion rate was included. Leukocyte counts on day 1 differed significantly between the no complications but also from the pneumonia cohort. This marker is established and is included in every routine laboratory. Furthermore, by including this parameter, valuable information about the immune homeostasis is added into the score. At last, the <200 nm EP rate has shown a clear correlation with sepsis development and was therefore included as novel directly cell-derived marker, including the wide range of small extracellular vesicles.

**Table 2:** Differences between the three cohorts in the relevant characteristics that were used for the calculation of the sepsis as trauma outcome prediction (STOP) score

Physiological and laboratory parameters	No complications (n = 79)	Pneumonia (n = 16)	sepsis (n = 29)	*P* < 0.05
**EP < 200 nm (number/μL) (IQR)**	14,904 (11652–20,520)	17,682 (12795–25,703)	12,138 (8198–16,472	b, c
**PRBC transfusion within 24 h (U)**	2.48 ± 1.14	1.50 ± 0.80	5.36 ± 1.70	b, c
**ISS (IQR)**	24.00 (18.00–29.00)	25.00 (17.00–41.00)	34.00 (23.50–38.50)	b
**Leukocytes day 1 (U/nL)**	9.46 (7.99–11.93)	11.34 (6.49–15.44)	6.95 (4.53–9.13)	b, c

Significant differences with *P* < 0.05 are indicating the following differences: no complication versus sepsis cohort (b), and pneumonia versus sepsis cohort (c).

EPs, extracellular particles; IQR, interquartile range; ISS, Injury Severity Score; PRBC, packed red blood cells.

**Fig. 3:**
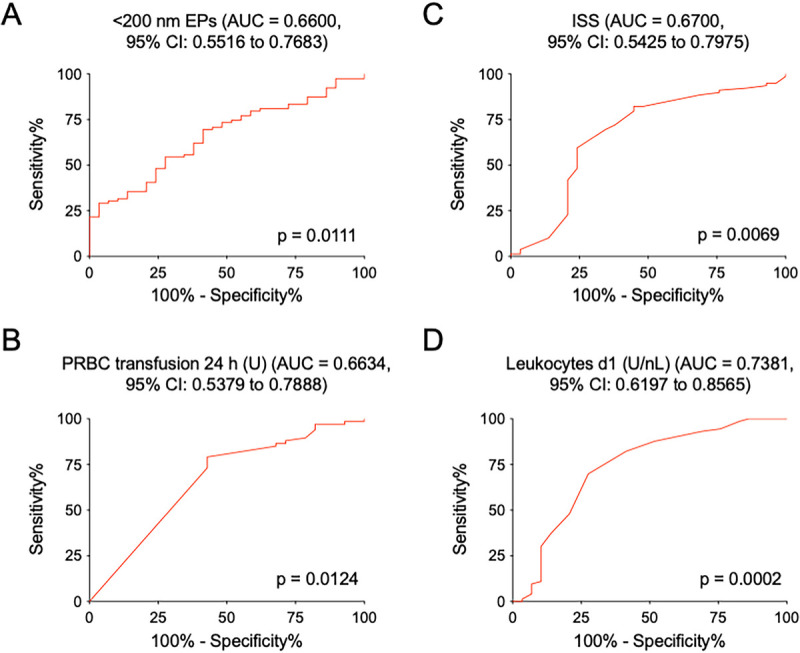
**Receiver Operating Characteristic curves with the AUC for sepsis-predictive variables: the EPs < 200 nm rate in the plasma upon admission (A), the PRBC transfusion rate within 24 h after admission (B), the ISS (C), and the leukocyte counts on day 1 after admission (D)**. AUC, area under the curve; CI, confidence interval; EPs, extracellular particle; ISS, Injury Severity Score; *P*, value of significance; PRBC, packed red blood cells; U, units.

ROC analyses were performed for all parameters to evaluate the prognostic performance of selected parameters for identifying patients at risk of sepsis development (Fig. 3). The ROC curve for <200 nm EPs at admission demonstrated an AUC of 0.6600 (95% CI: 0.5516–0.7683, *P* = 0.0111, Fig. 3A), indicating moderate predictive ability. A cutoff value of ≤12.639 yielded a sensitivity of 69.62% (95% CI: 58.25–79.47) and specificity of 58.62% (95% CI: 38.94–76.48), with a likelihood ratio of 1.682 (*P* = 0.0111, Table 3A).

**Table 3:** **Prognostic values and cutoff values for the rates of extracellular particles in the plasma upon admission, Injury Severity Score, packed red blood cells transfusion rate within 24 h after admission, and the leukocyte count on day 1 after admission to identify patients with development of sepsis (A). Additional results of the logistic regression analysis and the Wald-test (B)**. **The Positive Predictive Value (PPV), their sensitivity, specificity, and F1 score for the development of sepsis for patients with the according Sepsis as Trauma Outcome Prediction (STOP) Score (**C**).**

Table 3A						
Parameters (sepsis)	Cut-off value	Sensitivity % (95% CI)	Specificity % (95% CI)	AUC (95% CI)	Likelihood ratio	*P* < 0.05
**<200 nm EPs (number/μL)**	≤12,639	69.62 (58.25–79.47)	58.62 (38.94–76.48)	0.6600 (0.5516–0.7683)	1.682	0.0111
**ISS**	≥25.00	69.62 (58.25–79.47)	65.52 (45.67–82.06)	0.6700 (0.5425–0.7975)	2.019	0.0069
**PRBC transfusion within 24 h (U)**	≥1.00	79.10 (67.43–88.08)	57.14 (37.18–75.54)	0.6634 (0.5379–0.7888)	1.846	0.0124
**Leukocytes d1 (U/nL)**	≤8.50	69.86 (58.00–80.06)	72.41 (52.76–87.27)	0.7381 (0.6197–0.8565)	2.533	0.0002

**Table 3B**					
**Parameters (sepsis)**	**Coef.**	**z**	***P* > [z]**	**Significance**	**Odds ratio**
**<200 nm EPs (number/μL)**	−0.0001	−2.4432	0.0146	significant	0.9999
**ISS**	0.0395	1.4759	0.1399	not significant	1.0403
**PRBC transfusion within 24 h (U)**	0.0963	1.6736	0.0942	marginal	1.1011
**Leukocytes d1 (U/nL)**	−0.1447	−2.1092	0.0349	significant	0.8653
**Table 3C**					
**STOP value ≥**	**PPV**	**Sensitivity**	**Specificity**	**F1-score**	
**0**	0.234	1.0	0.0	0.379
**1**	0.267	0.931	0.221	0.415
**2**	0.333	0.931	0.432	0.491
**3**	0.471	0.828	0.716	0.6
**4**	0.594	0.655	0.863	0.623
**5**	0.643	0.31	0.947	0.419
**6**	0.75	0.207	0.979	0.324

The score was calculated by adding one point (for ISS and PRBC transfusion rate) or two points (for EP < 200 nm or Leukocyte count day 1) for each parameter, which was out of the cutoff values.

AUC, area under the receiver operating characteristic curve; CI, confidence interval; Coef., coefficient; EPs, extracellular particles; ISS, Injury Severity Score; PPV, positive predictive value; PRBC, packed red blood cells; STOP, Sepsis as Trauma Outcome Prediction; U, unit.

The ROC curve for PRBC transfusion within the first 24 h showed an AUC of 0.6634 (95% CI: 0.5379–0.7888, *P* = 0.0124, Fig. 3B), also suggesting moderate prognostic utility. PRBC transfusion within the first 24 h at a cutoff of ≥1.00 unit demonstrated a sensitivity of 79.10% (95% CI: 67.43–88.08) and specificity of 57.14% (95% CI: 37.18–75.54), with a likelihood ratio of 1.846 (*P* = 0.0124, Table 3A).

The ISS ROC curve yielded an AUC of 0.6700 (95% CI: 0.5425–0.7975, *P* = 0.0069, Fig. 3C), demonstrating a slightly better predictive value than the previous two parameters. The ISS with a cutoff value of ≥25.00 showed a sensitivity of 69.62% (95% CI: 58.25–79.47) and specificity of 65.52% (95% CI: 45.67–82.06), with a likelihood ratio of 2.019 (*P* = 0.0069, Table 3A).

The ROC curve for leukocyte counts on day 1 presented the highest AUC among the evaluated parameters, with an AUC of 0.7381 (95% CI: 0.6197–0.8565, *P* = 0.0002, Fig. 3D), indicating good prognostic capability. Leukocyte count on day 1 with a cutoff value of ≤8.50 U/nL showed the highest performance, with a sensitivity of 69.86% (95% CI: 58.00–80.06) and specificity of 72.41% (95% CI: 52.76–87.27), a likelihood ratio of 2.533 (*P* = 0.0002, Table 3A). Additional data on different cutoffs of the STOP score parameters are provided in supplementary Table 3.

To further evaluate the diagnostic value within the logistic regression analysis, we performed a Wald test, resulting in a significant correlation for EPs < 200 nm and leukocytes on day 1 (Table 3B). We further analyzed the diagnostic value for these two parameters only, which resulted in worse diagnostic values compared to the inclusion of ISS and the PRBC transfusion rate (Suppl. Table 4, http://links.lww.com/SHK/C454). Thus, we chose all four parameters to be included in the scoring. For the significant markers EP < 200 nm and leukocytes on day 1, two points were added to the Sepsis as Trauma Outcome Prediction (STOP) score, while for ISS and the PRBC transfusion rate one point was added, if a parameter met the defined cutoff.

Based on this scoring system, 8 patients (6.45% of all patients) achieved a STOP score of 6, of whom 6 belonged to the sepsis cohort, resulting in a positive predictive value (PPV) of 75% (Table 3C). A STOP score of 5 was calculated in 6 patients (4.84% of all patients), 3 of whom were in the sepsis cohort, leading to a PPV of 50%. In total 18 patients (14.52% of all patients) had a STOP score of 4, including 10 patients in the sepsis cohort, resulting in a PPV of 55.6%. Three points in the STOP Score were observed in 19 patients (15.32% of all patients), of whom 5 patients were in the sepsis cohort, leading to a PPV of 26.3%. Finally, 73 patients (58.87% of all patients) scored below 3 on the STOP scale, with only 5 patients in this group belonging to the sepsis cohort. This resulted in a PPV of 6.85% (Suppl. Table 5, http://links.lww.com/SHK/C455).

## DISCUSSION

Pneumonia and sepsis are among the most frequent complications in multiple-trauma patients (3). These infectious complications are largely attributed to the vast immunological reactions triggered by the traumatic injury as well as the patient- and injury-related characteristics and treatment strategies (2,3,25,26). However, the appropriate early assessment to guide therapeutic decisions is demanding because of the heterogeneity of multiple-trauma patients. While the current approaches rely on rapid assessment of various patient- and injury-related markers, they often exclude cell-derived factors (27). Therefore, we evaluated the prognostic value of a clinically fast implementable novel marker: (small) extracellular cell-derived particles (EPs) in plasma, in combination with routine markers, to identify patients at risk for pneumonia and sepsis.

### Assessment of pneumonia-related risk factors

Pneumonia is the most frequent infection after severe trauma (3,13), with established risk factors including age, male sex, intoxication, lower GCS, higher ISS, preexisting pulmonary diseases, chest trauma, transfusions, and prolonged mechanical ventilation (28–30). In our study, 12.9% (16 from 124) of patients developed pneumonia without concurrent sepsis. These patients had a significantly longer ICU stay, lower GCS in the field and at admission, elevated SOFA and APACHE-II scores at admission, higher rates of intubation in field and in the emergency department, and increased activated partial thromboplastin time compared to the no complication group.

A reduced GCS in the field, indicative for severe head injuries, emerged as a prominent risk factor. These injuries going along with severe alterations of the consciousness usually necessitate endotracheal intubation in the field (31), a known contributor to posttraumatic ventilator-associated pneumonia (32). Our study confirms this correlation between pneumonia development and the intubation rate. Furthermore, the continuously elevated SOFA and APACHE-II scores at admission and during the clinical course indicate preexisting organ dysfunctions as contributing factors. These findings align prior research linking advanced age and organ dysfunction to pneumonia risk in trauma patients (29).

Taken together, our study indicates patients with severe head injuries (low GCS) and preexisting organ dysfunctions (elevated SOFA and APACHE-II) are at higher risk of developing pneumonia. However, given the limited sample size and focus on patients without sepsis, these results must be interpreted carefully and require validation in larger patient cohorts.

### Assessment of sepsis-related risk factors

In our study cohort, 23.4% (29 out of 124) severely injured traumatized patients developed sepsis, a rate higher compared to other studies reporting 11.8% (23) to 13.8% (3). This discrepancy may result from our inclusion criteria, which excluded patients with immediate mortality. Lu *et al.* observed similar elevated sepsis rates of up to 30% by using coherent inclusion criteria (10). In the present study, several univariate risk factors associated with sepsis development compared to the no complication cohort were identified.

A higher ISS was significantly associated with prolonged need for mechanical respiration and delayed physical recovery (33), as well as hospital-acquired infections (34), and sepsis (9,23). Severe injuries are known to cause extensive tissue damage, promoting the release of immunomodulatory damage-associated molecular patterns (DAMP), which further trigger vast genomic changes in immune cells (4), and amplify their systemic activation, thus impairing their antimicrobial functions. For example, neutrophil granulocytes in trauma patients display an altered receptor expression, impairing chemotaxis, degranulation, and antigen recognition (35), while the monocytic phagocytic capacities are diminished (36).

Additionally, comprised barrier integrity resulting from immune activation can facilitate bacterial invasion, increasing infection risk (37). Interestingly, in our study, patients with sepsis exhibited significantly higher transfusions rates (PRBC and FFP) within the first 24 h after admission. Severe hemorrhage, which is not represented in the ISS calculation, correlates with the development of sepsis (9,38). While the underlying mechanisms are still elusive, severe hemorrhage, indicated by elevated transfusion needs, may exacerbate sepsis risk by increasing endothelial permeability (37), and glycocalyx shedding (39), thus compromising vascular integrity and facilitating pathogen invasion (40). These processes mimic mechanisms observed in sepsis itself (41), potentially creating a feedback loop that worsens outcomes.

Patients with severe thoracic trauma often experience significant blood loss, which is reflected by higher need of catecholamines, lower hemoglobin values, and higher transfusion needs upon admission (42). Similarly, in cases of severe abdominal trauma, blood loss is indicated by higher transfusion rates (43) or elevated initial lactate levels (44). In our study, specific injury patterns, notably chest and abdominal injuries were significantly more frequent in the sepsis cohort. The complication-promoting influence of severe chest injuries on sepsis has been demonstrated before in different studies (9,42,45). Clinical factors such as insufficient pain management (46) or suboptimal ventilation settings could further exacerbate the risk of infections and sepsis in mechanically ventilated patients (47).

Chest trauma, in particular, triggers profound immunological changes (48), including reduced concentration of circulating CD11c^+^ extracellular vesicles (49). First experimental therapeutical approaches, focusing on modifying the pulmonary inflammation after thoracic trauma, show promising results (50,51). To date, in patients with hemorrhage, only red blood cells, platelets and coagulation factors can be therapeutically substituted. In contrast, leukocytes must restore themselves to reestablish homeostatic ratio. In the present study, reduced leukocyte counts 1 day after trauma were associated with sepsis development. Early lymphopenia has been linked to multiple-organ dysfunction syndrome (MODS) after trauma, while persistent leukocytosis, conversely, has been described as a risk factor for MODS (52). Both findings highlight the importance of achieving an optimal early immune-cellular recovery and long-term homeostasis to avoid complications.

In this study, we demonstrated that not only the reduction of immune cells 1 day after trauma but also decreased levels of small cell-derived EPs at admission are valuable and significant markers for identifying patients at risk of sepsis during the clinical course following severe trauma. Extracellular particles encompass small, cell-derived structures including extracellular vesicles (EVs) and other particles of cellular origin. These particles have been increasingly studied as potential biomarkers due to their immunomodulatory cargo, including micro-ribonucleic acid and cytokines, and their surface marker profiles that resemble their originating cells. However, comparing findings on EVs and EPs across studies is challenging due to significant methodological differences in isolation, storage and characterization methods (22).

Our study utilized Calcein-AM staining to detect cell-derived particles in plasma, offering a simple, time-efficient, and cost-effective method for assessing the full spectrum of EPs without specific isolation procedures. Calcein-AM detects intact particles based on intracellular esterase activity, and the loss of signal after particle lysis, as described in our previous research and by others, supports its reliability for assessing intact EPs *via* the intravesicular signals (15,21). This fast approach in combination with a size differentiation provides a practical alternative for clinical use, especially in settings where complex isolation techniques may not be feasible. However, it is important to note that Calcein-AM is not specific for EVs. This limits the direct comparison of our results to other studies on EVs, which isolate and characterize EPs and especially extracellular vesicles using more sophisticated methodologies, as outlined in the latest MISEV guidelines (22).

Discrepancies in findings between EPs’ and EVs’ studies further highlight the need for standardization in future research. For instance, while our approach reliably detected small EPs, other studies using Calcein-AM reported an inability to detect larger EVs (>200 nm) (53). Conversely, other groups have successfully detected EVs using Calcein-AM staining (54,55). However, there are only few studies using Calcein-AM for EPs in multiple trauma patients. In a previous study following a similar approach, elevated rates of small EPs have been associated with increased in-hospital mortality in multiple-trauma patients (15). Despite the stated limitations on methodological comparison, analogous results have been observed for extracellular vesicles.

Dakhlallah *et al.* observed a trend to decreased EV counts in sepsis patients without septic shock compared to other critically ill patients, while septic shock nonsurvivors showed significantly increased rates of EVs compared to septic shock survivors and nonseptic critical ill patients (56), who had comparable rates of EVs in the plasma. Thus, EVs are considered as important biomarkers and regulators of sepsis (57).

The apparent contradiction between early elevated EPs correlating with in-hospital mortality and decreased EPs correlating with sepsis development points to a complex relationship between EV/EP dynamics and clinical outcomes and warrants further investigation. However, this paradox may be partially explained by the globally observed reduction in sepsis-related posttraumatic mortality rates (8). In our study, mortality rates between cohorts did not significantly differ, suggesting that sepsis development typically occurs later in the clinical course, while early trauma-related deaths are more closely related to injury severity (3).

This aligns with previous research emphasizing the importance of early interventions to prevent sepsis, which remains a significant complication with profound impacts on recovery and long-term quality of life (7). The clinical implication of our findings is substantial. Sepsis development in our study correlated with prolonged intensive care treatment and overall hospital stay, consistent with previous reports (58). Beyond its direct impact on physical health, sepsis is well-known to contribute to long-term impairments in mental health (59). These findings underscore the critical importance of preventing sepsis in trauma patients, not only to reduce in-hospital mortality but also to alleviate the long-term burden on patients and health-care systems.

### Patient assessment with the STOP score

The early recognition of hospitalized patients at risk of developing sepsis remains a critical focus in clinical treatment and research. Sepsis recognition in trauma patients presents unique challenges due to the overlap between trauma-related and SIRS-related organ dysfunctions and infection-induced dysfunctions. These distinctions are critical for meeting the diagnostic criteria consistent with the Sepsis-3 definition (13).

Automated logistic regression models designed to identify trauma patients at risk for sepsis show promise but lack specificity (60). While these approaches analyze a variety of variables, they are encouraging and show how large data sets after their precise implementation will help guide clinicians toward an individualized therapy, these models are still having several limitations in the emergency trauma surgery settings—where only limited patient data are available.

For sepsis patients, we identified several significant risk factors compared to the no complication cohort. To enhance early treatment decisions, we focused on variables measurable within the first 24 h after admission. These included: ISS as marker for injury severity, 24-h PRBC transfusion rate as key hemorrhage and coagulation parameter, leukocyte counts on day 1 after admission as basic immunological marker, and plasma rate of small cell-derived EPs as early, direct cell-derived factor. These values were combined to develop the STOP score. Given the results of the Wald test, we double-weighted the significant variables for the EP < 200 nm rate and the leukocyte counts on day 1.

Patients with a STOP score > 3 demonstrated a positive predictive value exceeding 50% for developing sepsis following trauma (Table 3C). The sensitivity of the STOP score >3 was 65.5% with a specificity of 86.3%. The STOP Score aligns with similar models, such as the Traumatic Sepsis Score (TSS) by Lu *et al.*, which also assesses parameters within the first 24 h after trauma. The TSS identified adult trauma patients in the age under 65 at risk for sepsis with a sensitivity of 64% and specificity of 82% (10). While the TSS includes ISS, GCS, temperature, heart rate, albumin, INR, and CRP, the STOP Score provides comparable sensitivity (65.5%) and specificity (86.3%) with fulfilling the criterion STOP score >3. Thus, the STOP Score focuses on a more concise set of variables. The biggest advantage of the STOP score lies in identifying patients at high risk for a sepsis at an early stage, enabling targeted therapeutic considerations. By stratifying patients at high risk for sepsis, patient enrollment criteria for clinical trials, for example, such as those assessing the efficacy of antioxidative effects of vitamin C therapy (61) could be refined. Recent studies focus on restoring the lymphocyte fraction in septic shock using IL-7, while the potential in severely multiple traumatized patients is not known (62). The STOP score could act as a screening tool for high-risk multiple trauma patients, potentially responsive for such novel immunomodulatory therapies. Additionally, by the initially identification of patients at high risk for sepsis, serially measured key diagnostic markers could be allocated and interpreted more precisely, potentially enabling faster therapeutic measurements, which are crucial in the care of septic patients.

Further potential fields of the STOP score, after validation in a larger patient cohort, could lie in different prophylactic strategies, including antibiotic administration or fluid substitution protocols.

Given the profound impact of sepsis on long-term outcomes and quality of life in trauma patients, we are confident that the STOP score represents a promising tool for early identification and risk stratification. By enabling individualized therapeutic strategies, it holds the potential to significantly improve sepsis outcomes and reduce its occurrence in trauma populations. Future studies are essential to confirm its utility and expand its applications in broader clinical settings.

## CONCLUSIONS

The study identifies the main risk factors for pneumonia without sepsis development in adult patients following severe multiple trauma as decreased Glasgow Coma Scale and higher intubation rates in the field, and besides prevalent organ dysfunctions at admission. For the development of sepsis after severe multiple trauma, significant risk factors in the logistic regression included a leukocyte count ≤8.5 U/nL 1 day after admission and a small EP concentration in plasma at admission ≤12.639 U/μL. These factors were double-weighted. We further included the significant univariate factors of an ISS ≥ 25, and receiving ≥1 transfusion during the first 24 h after admission. Patients having more than three points in the weighted STOP score have a risk exceeding 50% for developing sepsis during their clinical course.

We suggest assessing multiple trauma patients using the STOP Score to identify those at risk for sepsis and to be considered for treatment and prevention refinements, after an external validation.

Given the significant impact on long-term outcomes and quality of life, the inclusion of cell-derived factors into the STOP score risk assessment represents a promising tool for more individualized therapeutic approaches. Future studies are essential to validate its utility and expand its clinical applications.

### Limitations

This study acknowledges several limitations that may influence the interpretation of its findings. Although patient inclusion was conducted prospectively, sample processing and data analysis were performed retrospectively without a validation cohort. Before the clinical implementation of the STOP score, we emphasize the need for a larger, multicenter validation study. Based on the acquisition method of the EP rates, cutoffs are particularly susceptible to vary between different trauma-centers. Moreover, interpretation of the clinical course data must account for the fact that only patients who survived the initial trauma were analyzed. The exclusion of patients on preexisting anticoagulative medication further limits the applicability of our findings to a specific patient cohort. It should be noted that a certain bias in the inclusion process is possible, as in cases of septic patients, a more prolonged period was often available to obtain consent from the patient or their legally authorized representative. This may explain their increased numbers compared to other groups.

Another limitation is the lack of data on specific causes of death across the cohorts. While we observed mean survival times of 4.75 ± 1.15 days (no complications, n = 12), and 4.67 ± 0.67 days (pneumonia), the only patient who died in the sepsis survived 14 days (n = 1), the absence of cause-specific mortality limits our ability to distinguish between trauma-related and complication-related deaths. This distinction is clinically relevant, as prior work has shown early deaths are typically trauma-related, while later deaths are often due to complications (3). Another limitation is that neither platelet transfusions nor crystalloid infusions can be reported. The retrospective design and reliance on the highest SOFA score within a 24-h period, while pragmatic, lack the granularity of continuous monitoring achievable in prospective studies. As highlighted by Kim *et al.* (63), the initial SOFA score at sepsis recognition has lower prognostic accuracy compared to the 24-h maximal SOFA score in emergency department patients with septic shock. Their findings emphasize the importance of continuous SOFA score monitoring to better capture the dynamic progression of sepsis. Although this study demonstrates that the 24-h maximal SOFA score provides meaningful and clinically relevant data, prospective studies with continuous SOFA monitoring are essential for a more comprehensive understanding of sepsis progression. A limitation of our study is the inclusion of patients with both pneumonia and sepsis in the sepsis group, which may have introduced confounding and reduced the specificity of SOFA score dynamics, potentially contributing to the unexpected finding that SOFA changes predicted pneumonia but not sepsis. Future investigations should aim to validate these findings in a larger, more diverse patient population, enabling subgroup analyses to assess the potential influence of medication on our findings. Furthermore, a multicenter approach is also necessary to assess variability in the recognition of EPs in plasma, which may arise due to differences in measurement devices. It must be noted that the consumable costs of this EPs test are lower than those of a standard ELISA; however, it requires access to a flow cytometer, which depending on the model and capabilities is linked to higher costs, and the method is not yet an approved clinical test routinely available in hospital laboratories. Standardizing methods for both EP and vesicle isolation, storage, and characterization will be critical to improving comparability across studies. While the methods employed in this study are practical for broad clinical applications, more specific techniques could provide deeper insights into extracellular vesicle subtypes and their roles in trauma and sepsis. Further research is needed to elucidate the biological mechanisms underlying the dynamics of EPs and their associations with sepsis progression and mortality. Prospective, multi-institutional studies that integrate continuous monitoring and standardized methodologies will be vital to advancing the clinical understanding and applicability of these findings.
